# The influence of different composite placement techniques on microleakage in preparations with high C- factor: An *in vitro* study

**DOI:** 10.4103/0972-0707.45249

**Published:** 2008

**Authors:** Lekha Santhosh, Kusum Bashetty, Gururaj Nadig

**Affiliations:** The Oxford Dental College and Hospital, Bommanahalli, Bangalore, Karnataka, India

**Keywords:** C-factor, composite placement techniques, microleakage

## Abstract

**Objective:**

This study evaluated the marginal leakage around class-I cavity preparations restored with Nanofilled composite (Filtek Z-350 A2 shade, 3M ESPE, USA) and a self-etch adhesive (Xeno III, DENTSPLY/Caulk) using different composite placement techniques.

**Materials and Methods:**

Standardized class-I cavities were prepared on 36 caries-free, extracted human premolars and were randomly assigned to three groups: (1) Horizontal incremental curing was done; each increment of thickness 1.5 mm was cured one after the other using curing unit (T-LED, Elca Technology, Italy). (2) Concave surface was obtained with a ball burnisher on the first increment and cured for 20 seconds; subsequently, the next increment was placed and similarly cured. (3) Cavities were filled with resin, short of the occlusal surface; two cuts (mesiodistal and buccolingual) were made through the condensed resin and cured for 20 seconds, followed by addition of resin in the gaps created by the cuts and additional curing for 20 seconds. The specimens were stored in distilled water for three months and then subjected to thermocycling, followed by immersion in 0.5% methylene blue dye for 24 hours. The teeth were sectioned longitudinally and evaluated for microleakage under stereomicroscope, and the scores obtained were analysed with Fisher Exact test and Kruskal-Wallis nonparametric test.

**Results:**

There was no statistically significant difference among three groups.

**Conclusion:**

None of the techniques was capable of eliminating the microleakage in preparations with a high C-factor.

## INTRODUCTION

Resin composite restorations have gained popularity because they match the shade of the natural teeth, are mercury-free and thermally non-conductive,[1] and they bond to the tooth structure with the use of adhesive agents.[2] Although composites are now the material of choice for most restoration, their polymerisation shrinkage remains a problem.

Modern resin composites undergo volumetric contractions of between 2.6 and 7.1%,[3] resulting in shrinkage stress generation at the composite-tooth interface.[4] These stresses may cause the composite to pull away from the cavity margins, resulting in adhesive failure and marginal gap formation.[5] Oral fluids containing bacteria may fill these gaps, causing microleakage and secondary caries. Other adverse consequences of shrinkage stresses include coronal deformation resulting in postoperative sensitivity, propagation of existing enamel microcracks, and microcracks of composite resin due to cohesive failure.[4]

In 1987, Feilzer *et al*. postulated that the geometric configuration plays an important role in the adaptation of resin composite restoration.[6] The cavity configuration (C-factor) is defined as the ratio of bonded to unbonded surfaces. A high ratio denotes high polymerization stresses, which are accompanied by increased shrinkage stresses. Among many of the factors contributing to the shrinkage stresses, C-factor is an important one.

Several techniques have been suggested to improve marginal adaptation of high C-factor preparation, including adhesive systems that potentially resist composite shrinkage,[7,8] placement techniques for resin composites,[9,10] protocols for polymerisation[11] and different cavity preparations.[5,12]

The purpose of the present *in vitro* study was to evaluate microleakage around Class-I resin composite restoration in preparations with high C-factor. Different placement techniques were designed to minimize the C-factor, which may lead to a decrease in the polymerization shrinkage stresses generated and, as a result, reduce the marginal gap formation.

## MATERIALS AND METHODS

Thirty-six extracted noncarious human premolars without enamel fracture were cleaned and stored in saline solution (0.9%) at room temperature. Occlusal surfaces were ground with a coarse diamond bur, under profuse water cooling, to produce a flat surface perpendicular to the long axis of the tooth, without removing whole of the occlusal enamel. Class-I cavity preparation of approximately 3 mm in length, 2 mm in width and 3 mm in depth was prepared using straight fissure bur (FG 111 012, Horico, Germany), with a high speed handpiece and copious amount of water. No bevels were placed.

All teeth were restored with the same adhesive system (Xeno III), according to the manufacturer's instructions and with the same restorative material (Filtek Z-350 A2 Shade). To light cure the composite resin, a curing unit (T-LED, Elca Technology, Italy) was used, set to a light intensity of approximately 800 mW/cm^2^. The specimens were divided into three experimental groups, with 12 teeth each, and the restorative material was inserted and hand condensed into the cavity preparation, according to the following placement techniques:

*Group-I:* The first increment of thickness 1.5 mm was inserted in a horizontal direction and cured for 20 seconds, followed by placement of the second increment of the same thickness and similarly light cured.

*Group-II:* The first increment of 2 mm thickness was inserted and a ball burnisher was used in a rocking motion to spread the resin. A concavity was created and then cured for 20 seconds. The second increment was inserted to fill the cavity and cured for 20 seconds.

*Group-III:* The cavity was filled with resin, short of the occlusal surface and two cuts (mesiodistal and buccolingual) were made with a Teflon coated plastic instrument (Hu-Friedy composite placement instrument), through the condensed resin, and cured for 20 seconds. Each cut extended down to the entire cavity depth. The second increment was inserted to fill these gaps and further cured for 20 seconds.

Immediately after curing, each restoration was contoured with finishing burs operated at high speed, using air-water coolant. All preparation, restoration and finishing were carried out by one author simulating clinical instrumentation, as much as possible. After finishing/polishing, the teeth were stored in distilled water at room temperature (30°C-36°C) for three months and then were subjected to 1000 thermal cycles between 5° and 15°C water baths. Dwell time was one minute, with a five second transit time between baths.

After thermocycling, the apices of the teeth were sealed with acrylic and all tooth surfaces, except for a 1 mm wide zone around the margins of each restoration, were sealed with two coats of nail polish. The teeth were then immersed for 24 hours in a 0.5% solution of methylene blue dye. The teeth were rinsed and then sectioned longitudinally in a mesio-distal direction, coincident with the centre of the restoration, using slow speed diamond disc cooled with water. The two hemisections of each tooth showing the cleanest dye penetration was selected and examined at 20X magnification, under a Stereomicroscope (Lawrence and Mayo, Labomed Zoomer). The degree of leakage was observed and scores were given according to an ordinal ranking system (0-4), as shown in Table 1.

**Table 1:** Criteria scores

Score	Criteria
0	No evidence of dye penetration at the tooth restoration interface
1	Dye penetration along the cavity wall, up to 1/3rd of the cavity depth
2	Penetration > 1/3rd but < 2/3rd of the cavity depth
3	Penetration > 2/3rd of the cavity depth, but not along the dentinal tubules
4	Penetration to cavity depth and along the dentinal tubules

The data were subjected to 2×3 Fisher Exact test, to check the percentage of microleakage in each criterion, and Kruskal-Wallis test, to compare the differences among the groups (*P*<0.05).

## RESULTS

**Figure 1:**
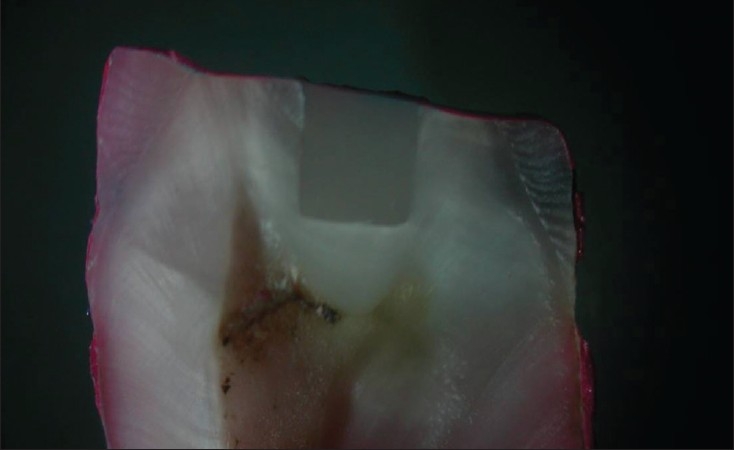
Group I – Exhibiting leakage

**Figure 2:**
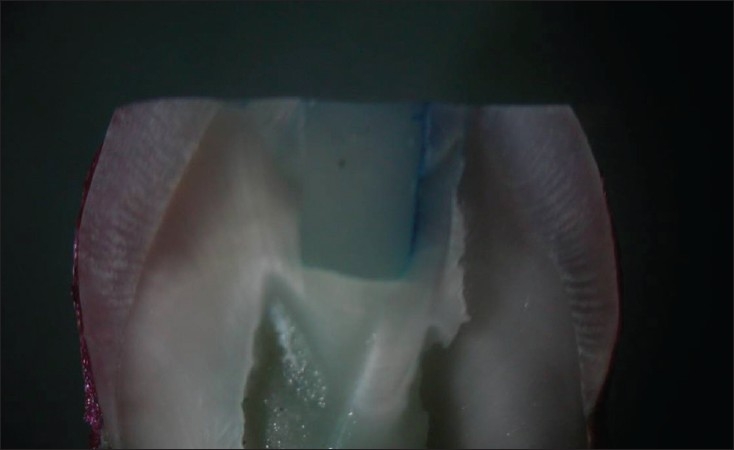
Group II – Exhibiting leakage

**Figure 3:**
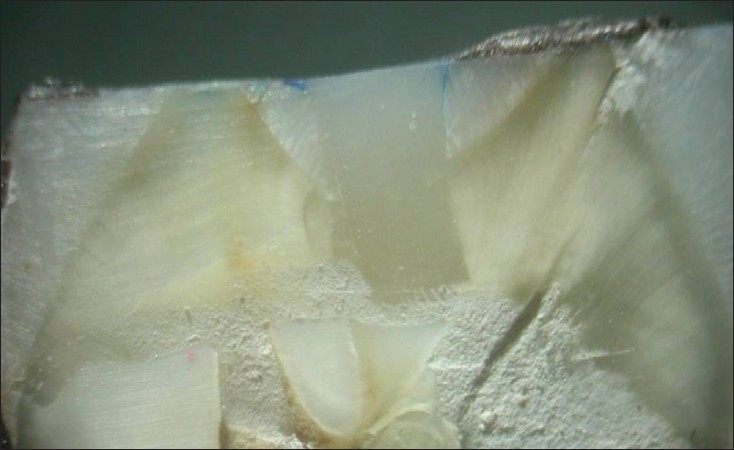
Group III – Exhibiting leakage

Table 2 displays the microleakage scores for the three resin composite placement techniques and Figures 1-3 shows microleakage in different groups. The Kruskal-Wallis test for the comparison of placement techniques found no statistically significant difference among the three experimental groups (*P*>0.4).

**Table 2:** Microleakage scores

Groups	Leakage scores	Total
	0 1 2 3 4	
I	1 6 3 2 0	12
II	3 7 0 1 1	12
III	4 4 2 2 0	12
TOTAL	8 17 5 5 1	36

## DISCUSSION

The service life of a resin composite restoration is dependent on several factors, including the cavity-composite interface sealing.[13] From this view point, investigations of factors related to gap formation mechanism are crucial in improving the clinical longevity of resin composite restorations.

Control of polymerization shrinkage stresses during a direct composite resin restoration is important for achieving a perfect adaptation between restoration and cavity wall. In order of importance, the factors involved in shrinkage stresses are cavity C-factor, cavity size, application technique for placement of composite, intensity and position of curing light, and properties of composite. In this study, attempts were made to keep all these variables constant, except for the composite placement technique. The different placement techniques used were horizontal incremental technique, horizontal increment with use of ball burnisher to form a concave surface and split technique. Class I cavities with depth of 3 mm were made due to high C factor ratio that causes higher polymerization stresses as a result of restrained contraction by the bonded surface. Class I cavities were used in preference to class V, as depth of 3 mm would result in pulpal exposure in the latter.

Studies have reported that with the horizontal technique, each composite increment that connects the occlusal cavity floor with the four surrounding walls produces the highest and most unfavourable C-factor ratio of 5. This consequently produces the highest shrinkage stresses in between the opposing cavity walls.[14] By contrast, it was anticipated that the proposed ball burnisher technique and split technique would decrease the C-factor and the shrinkage stresses between the opposing cavity walls. In Group II, a concavity was created with a ball burnisher on the first increment, in order to increase the surface area of unbonded surface, thus decreasing the C factor, shrinkage stress and subsequently the microleakage. In this study, though the microleakage scores in Group II were less, when compared to group I, it was not statistically significant.

In Group III, two perpendicular cuts were made in the condensed composite before curing, in order to avoid the composite being in contact with opposing cavity walls. The free unbonded composite surface thus created by the cuts would have converted the restricted shrinkage to unrestricted shrinkage. In the initial stage of polymerization, these free composite surface would act as a reservoir for the flow or plastic deformation and minimize the shrinkage stresses.[15] There was less microleakage in Group III, as compared to Group I, but there was no statistical significance. The reason could be that though the splits were made to floor level, they were not complete due to the flow of the composite. Horizontal increments filled up to half of the cavity depth with splits and use of condensable composites may have given a favourable result. Diagonal cuts resulting in triangular portions should also be studied.

Based on these results, it can be inferred that for class-I preparations to be restored with composite resin, the adhesive system (Xeno III) employed and the nanofilled composite resin used were capable of generating an effective bonding at the tooth/restoration interface, regardless of the restorative technique utilized. Xeno-III contains nanofillers. Previous findings[16,17] have reported that the collagen fibril network mostly filters out nanofillers, holding them at the hybrid layer surface, thus acting as an intermediate shock absorber. A reduced microleakage score has been reported when using filled adhesives.[18,19] Nanofilled composite resin was used in this study, as the filler particle diameter was about half the wavelength of the activating light and the light scattering was increased, thereby decreasing the degree of conversion and consequently polymerization shrinkage.[20]

Duarte and Dinelli,[21] and Sensi[22] found no significant marginal leakage improvement when restored with increment placement and bulk placement technique in class V preparations. Neiva *et al*.[23] have shown that there is no statistical significance in marginal leakage in class II restorations when the gingival margin is in enamel, using split increment technique.

This study adapted a combination of two commonly used ageing processes to simulate the degradation of bond over time, in the oral cavity, i.e. ageing by storage and ageing by thermocycling. It may be speculated that a combination of the two processes can increase the effect on artificial ageing, thus increasing microleakage.

Marginal integrity and microleakage *in vitro* experiments are currently being performed to evaluate the effects of the different placement techniques on the quality of the margins in composite restorations. This study was performed *in vitro*, which can be a screening procedure for ensuing *in vivo* studies. Previous studies have indicated that data obtained from *in vitro* microleakage testing may be useful, but not always necessarily reproducible in clinical *in vivo* settings.[24] Also, in performing *in vitro* microleakage investigations, obtaining conclusive information can be problematic, since vast differences in research protocols are reported in the dental literature; leakage patterns are highly complicated and irregular; and, one section of the tooth cannot be relied on for drawing a conclusion. Further studies are required before definite conclusions can be formulated.

## CONCLUSION

None of the techniques for resin placement was able to eliminate marginal microleakage in Class-I cavity preparation. There was no statistical difference among the three groups with different resin placement techniques in cavity with high C-factor. The control of marginal microleakage with a high C-factor presents a challenge, regardless of the resin composite insertion techniques.
